# Optimisation Modelling to Assess Cost of Dietary Improvement in Remote Aboriginal Australia

**DOI:** 10.1371/journal.pone.0083587

**Published:** 2013-12-31

**Authors:** Julie Brimblecombe, Megan Ferguson, Selma C. Liberato, Kerin O'Dea, Malcolm Riley

**Affiliations:** 1 Menzies School of Health Research, Darwin, Northern Territory, Australia; 2 Division of Health Sciences (School of Population Health), University of South Australia, Adelaide, South Australia, Australia; 3 Commonwealth Scientific Industrial Research Organisation (Animal, Food and Health Sciences), Parkville, Victoria, Australia; The Ohio State University, United States of America

## Abstract

**Background:**

The cost and dietary choices required to fulfil nutrient recommendations defined nationally, need investigation, particularly for disadvantaged populations.

**Objective:**

We used optimisation modelling to examine the dietary change required to achieve nutrient requirements at minimum cost for an Aboriginal population in remote Australia, using where possible minimally-processed whole foods.

**Design:**

A twelve month cross-section of population-level purchased food, food price and nutrient content data was used as the baseline. Relative amounts from 34 food group categories were varied to achieve specific energy and nutrient density goals at minimum cost while meeting model constraints intended to minimise deviation from the purchased diet.

**Results:**

Simultaneous achievement of all nutrient goals was not feasible. The two most successful models (A & B) met all nutrient targets except sodium (146.2% and 148.9% of the respective target) and saturated fat (12.0% and 11.7% of energy). Model A was achieved with 3.2% lower cost than the baseline diet (which cost approximately AUD$13.01/person/day) and Model B at 7.8% lower cost but with a reduction in energy of 4.4%. Both models required very large reductions in sugar sweetened beverages (−90%) and refined cereals (−90%) and an approximate four-fold increase in vegetables, fruit, dairy foods, eggs, fish and seafood, and wholegrain cereals.

**Conclusion:**

This modelling approach suggested population level dietary recommendations at minimal cost based on the baseline purchased diet. Large shifts in diet in remote Aboriginal Australian populations are needed to achieve national nutrient targets. The modeling approach used was not able to meet all nutrient targets at less than current food expenditure.

## Introduction

Similar to that of other low income populations, the contemporary diet of Aboriginal Australians has been characterised as excessive in refined carbohydrate, sodium, and low in fruit and vegetables[1]; [2]. High food costs and low socio-economic position are key determinants of this poor quality diet[3]. Surveys to assess the cost of a defined basket of foods in different states[4]; [5] and the Northern Territory (NT) of Australia[6] have consistently demonstrated relatively higher food costs outside major cities that increase with categories of remoteness[4]. The cost is 49% higher for remote NT communities compared to a provincial city supermarket[6]. Poor quality diets are associated with low socio-economic position in Australia[7]–[9] and other developed countries[10]; [11]. This is particularly significant for Indigenous Australians, where past estimates reveal nearly one-third (30.8%) of Aboriginal households in the severe poverty category[12].

Approaches to improve nutrition for Aboriginal populations in remote Australia aim to modify dietary behaviour to be consistent with national level dietary recommendations. To our knowledge the possibility of providing an affordable healthy diet that meets dietary recommendations, based on a combination of foods currently consumed, has not been determined for the general Australian population nor for Indigenous Australians living in remote communities. Nutrition education strategies suggest replacing less healthy foods with healthier options, such as consuming less refined carbohydrates and more fruit and vegetables, or substituting whole-wheat bread for white bread. Whether these dietary recommendations cost more than current food expenditure is contentious. Assessments comparing the cost of a healthier basket of foods to a standard food basket, where healthier food types are directly substituted for a standard option (such as reduced fat milk for regular milk), have in some instances been shown to be more costly[13], of similar price[14] and in other cases less costly[15]. A major concern for inference from this method of direct substitution is the failure to correct for the likely difference in energy content of the baskets[16]. Moreover the basket of foods may not reflect the food preferences or eating habits of the population[17]. Specially tailored meal plans to accommodate limited food budgets are often limited in variety[18], demand home food preparation and adequate food storage facilities (which are severely lacking in many remote Aboriginal communities and include much less fresh produce[17]. On the basis of self-selected diets, it has been shown that high-quality diets cost more under normal circumstances[19]–[21] and higher diet costs have been associated with consuming more fruit and vegetables and less energy from fat, alcoholic beverages and added sugars[22].

Optimisation modelling has been used to identify the dietary changes required for achieving nutrient recommendations, while deviating the least from population dietary habits[15]; [17]; [23]–[26]. The aim of this study was to investigate the effect of applying nutrient recommendations on the structure and cost of the diet of an Aboriginal population in remote Australia, using optimisation modelling with minimum cost as the objective function.

## Materials and Methods

This study was approved by the Human Research Ethics Committee of the Northern Territory Department of Health and Menzies School of Health Research and the Central Australian Human Research Ethics Committee. All participating stores and providers of food services gave written consent.

### Sampling of communities

Convenience sampling was used to select three remote communities that were then invited to participate in the study. These communities were located in the Northern Territory, had previously provided electronic point-of-sale data for research, and were characteristic of the variation in size, distance from a metropolitan centre and number of food businesses and services observed in remote Aboriginal communities[1].

### Setting

The three communities had a combined population estimated at 2644 residents of mostly Aboriginal ethnicity and 34–41% of residents <18 years of age[1], were located in both Central and Coastal NT, and were classified as very remote in reference to very little accessibility of goods and services[27]. Distance from each community to the nearest food wholesaler ranged from 130 km to 520 km. Alcohol was not available for purchase in the study communities at the time of the study. These communities, as in most Aboriginal communities in remote Australia, had a small store as the primary food outlet and food services for school-aged children (which may be a school canteen or served meals) and the aged. Traditional foods are also procured. Food was primarily sourced from the store and food services for the three study communities[1].

Current population data were not available at the time of the study. To report per capita dietary cost and energy intake, the population of the three communities combined was estimated based on total energy available in the food supply (2010/11) relative to a weighted energy requirement for the population distribution. Detailed methods for determining population have been described elsewhere[1]. The weighted energy requirement for the study population was derived using the estimated energy requirement as stated in the Nutrient Reference Values for Australia and New Zealand[28] (using a physical activity factor of 1.6 (National Health and Medical Research Council (NHMRC) – light activity) for each age group and sex[29], in conjunction with the population age and sex distribution as determined by the 2006 ABS population census for each of these three communities[30]. This derived total population was checked with Australian Bureau of Statistics (ABS) estimates[31].

### Data collection

Monthly electronic food transaction data were provided by stores and food order data were collected from food suppliers for all food services (including school canteens, meals for elderly, and school breakfast/lunch programs) in each of the three communities for the period July 2010 to June 2011. All food and beverage items with their accompanying Universal Product Code or store derived product code, the quantity sold, and the dollar value (retail price) were imported to a purpose designed Microsoft Access database[32] and linked to Australian Food and Nutrient Data (AUSNUT 2007, NUTTAB 06 and AUSNUT 1999)[33]–[36] with amendments for folic acid using NUTTAB 2010[34]. Food data were categorised into 34 food groups derived from groups developed for the modelling system to inform the revision of the Australian Guide to Healthy Eating (AGHE)[37] (Table l). Two subcategories were defined for most food groups (e.g., refined cereals) which identified foods to be encouraged and designated “IN” (e.g., white bread, pasta, rice) or foods to be discouraged and designated “OUT” (e.g., sweet biscuits, pastry, cakes) (Table 2).

**Table 1:** Food Groups.

Modelling Food Group	AUSNUT food sub-groups associated with Food Group	Examples of “IN” category
Artificial sweeteners,/diet products (no calories)	Artificial sweeteners	Not applicable (NA)
Beverages	Fruit drinks, cordials, soft drinks, fruit and vegetable juices, juice drinks, teas, coffees, dry beverages flavourings	Tea, coffee, water and artificially sweetened beverages
	Beers, wines, other alcoholic beverages	Not available in study communities
Cooking additives	Additives and cooking ingredients	NA
Dairy foods	Butters, cheese products, creams, frozen milk products where milk is major component, imitation dairy products, low fat & fat modified cheeses, milk, condensed, milk, fluid, other dishes where milk is major component, traditional cheese, yoghurt full fat, yoghurt low fat	Low fat & fat modified cheeses, unsweetened/low fat yoghurt, reduced fat milk (plain - fluid and powdered)
Eggs	Egg, eggs substitutes, egg dishes where egg is major component	All included as “IN”
Fats and oils	Poly margarines, other margarines, vegetable oils and Other fats	Canola margarine, canola oil, reduced salt margarines, olive oil, sunflower seed oil, monounsaturated margarines
Fish and seafood	Fin fish, other sea & freshwater foods, crustacea & molluscs products & dishes	Sardines, fresh and frozen fish, fresh and frozen seafood, tuna, tinned oysters, tinned mussels
Fruit	Packing liquid processed berry fruit, packing liquid processed citrus fruit, packing liquid processed stone fruit, packing liquid processed other fruit, composite^1^ fruit product where fruit is major component, packing liquid processed composite fruits, berry fruit, citrus fruit, stone fruit and other fruits	All included as “IN”
Green and brassica vegetables	Vegetable, mature legumes, composite food where mature vegetable is major component, composite food where mature legume is major component	Fresh and frozen vegetables, reduced salt tinned vegetables (Na <300 mg/100 g)
Infant products	Infant cereals, infant rusks and fingers, infant dinners strained junior and toddler, infant fruit and deserts, infant fruit juices	NA
Legumes	Mature legumes, composite food where mature legume is major component	All included as “IN”
Nuts and seeds	Seeds and seed products, nuts & nut products	Unsalted products
Orange vegetables	Vegetables, mature legumes, composite food where vegetable is major component, composite food where mature legume is major component	Fresh and frozen vegetables
Other vegetables	Vegetables, mature legumes, composite food where vegetable is major component, composite food where mature legume is major component	Fresh and frozen vegetables
Poultry	Poultry, feathered game	Fresh and frozen chicken and other birds
Red meats	Beef, lamb, pork, veal, game and other carcass meats, feathered game, offal & offal products, Battered and crumbed products, sausages, frankfurts, saveloys, other processed meats, meat pastes, Composite meat & poultry products where meat is a major component, vegetarian meat substitutes	Fresh and frozen beef, lamb, pork, game, offal
Refined cereals	Breads rolls, grains & starches, flours, muffins crumpets, other cereal-based bread equivalents, savoury biscuits, sweet biscuits, ready-to-eat breakfast cereals, cooked breakfast cereals, cakes, muffins, puddings, buns, scones, batters, pastries, sweet pastry products, savoury pastry products, pizza, sandwiches, filled rolls, other products where cereal is major component	Rice, fresh noodles, flour, white bread, pizza bases, hamburger rolls, breadcrumbs, white pita bread, sandwiches, muffins, tortillas, rice crackers, rice snacks, rice cakes, spaghetti, pasta, macaroni, rice bubbles,
Sauces and condiments	Sweet sauces, savoury sauces, pickles, soups, snack foods, herbs & spices, vinegars, salad dressings, yeast, yeast vegetable extracts, essences, others	All included as “OUT”
Starchy vegetables	Vegetables, mature legumes, composite food where vegetable is major component, composite food where mature legume is major component	Fresh vegetables, frozen vegetables (eg excluded potato chip products)
Sweets	Sugars, preserves, confectionery and composite foods where sugar is major component	Artificially sweetened “sweets”
Wholegrain cereals	Breads rolls, grains & starches, flours, muffins crumpets, other cereal-based bread equivalents, savoury biscuits, sweet biscuits, ready-to-eat breakfast cereals, cooked breakfast cereals, cakes, muffins, puddings, buns, scones, batters, pastries, sweet pastry products, savoury pastry products, pizza, sandwiches, filled rolls, other products where cereal is major component	All included as “IN”

^1^ The term “composite” refers to a mix of ingredients

**Table 2:** Model constraints set for change in food group weight expressed as a multiple of 12 month food group total volume of the communities.

Food Group	Multiple of baseline intake
1. Artificial sweeteners	= 1
2. Beverages (“IN”)	≥0.5 to ≤4
3. Beverages (“OUT”)	≥0.1 to ≤0.5
4. Cooking additives	= 1
5. Dairy foods (“IN”)	≥1 to ≤4
6. Dairy foods (“OUT”)	≥0.5 to ≤4
7. Eggs	≥1 to ≤4
8. Fats (“IN”)	≥1 to ≤4
9. Fats (“OUT”)	≥0.5 to ≤1
10. Fish and seafood (“IN”)	≥1 to ≤4
11. Fish and seafood (“OUT”)	≥0.5 to ≤1
12. Fruit	≥1 to ≤4
13. Green and brassica vegetables (“IN”)	≥1 to ≤4
14. Green and brassica vegetables (“OUT”)	≥0.5 to ≤1
15. Infant products	= 1
16. Legumes	≥1 to ≤4
17. Nuts and seeds (“IN”)	≥1 to ≤4
18. Nuts and seeds (“OUT”)	≥0.5 to ≤1
19. Orange vegetables (“IN”)	≥1 to ≤4
20. Orange vegetables (“OUT”)	≥0.5 to ≤1
21. Other vegetables (“IN”)	≥1 to ≤4
22. Other vegetables (“OUT”)	≥0.5 to ≤1
23. Poultry (“IN”)	≥1 to ≤4
24. Poultry (“OUT”)	≥0.5 to ≤1
25. Red meats (“IN”)	≥1 to ≤4
26. Red meats (“OUT”)	≥0.5 to ≤1
27. Refined cereals (“IN”)	≥0.5 to ≤0.7
28. Refined cereals (“OUT”)	≥0.1 to ≤0.5
29. Sauces and condiments	≥0.5 to ≤1
30. Starchy vegetables (“IN”)	≥1 to ≤4
31. Starchy vegetables (“OUT”)	≥0.5 to ≤1
32. Sweets (“IN”)	≥0.5 to ≤1
33. Sweets (“OUT”)	≥0.5 to ≤1
34. Wholegrain cereals	≥1 to ≤4

For each food group, total edible weight (adjusted for specific gravity[36]) and total nutrient composition (i.e., the total energy in kJ and total nutrient content in grams) were determined by the sum of the individual foods that were categorised into that group and for the three communities combined (community diet). Nutrient density was calculated for each food group as nutrient amount divided by energy contributed by the food group. Nutrients examined were protein, total fat, saturated fatty acids, carbohydrates, total sugars, long chain fatty acids, α-linolenic and linoleic fatty acids; and vitamin A, thiamine, niacin equivalents, riboflavin, vitamin E α-tocopherol equivalents, vitamin C, total folate, magnesium, iodine, phosphorus, zinc, potassium, calcium, sodium and iron. Dietary fibre was also examined.

### Dietary Modelling

An optimisation model is an objective function dependent on a set of decision variables subject to a number of constraints[24]; [38]; [39]. The optimisation goal is to find those values for the set of decision variables that produce the best value for the objective function while meeting all the imposed constraints. In the present study, the goal is to minimise total dietary cost to communities by modification of the amount consumed from a range of food groups, subject to two sets of constraints - one relating to nutrient adequacy of the diet and expressed through nutrient density, and the other set relating to food group intake. The constraints relating to nutrient adequacy were intended to ensure that the modelled dietary intake at community level was consistent with nutrient adequacy, while the constraints relating to food group intake were intended to result in total dietary intake that represented a broadly pragmatic change from current dietary intake. Optimisation results in either a feasible solution (optimised objective function) or a solution cannot be found given the constraints (meeting all of the constraints is not possible). In this case, constraints can be removed or modified to be less restrictive in order to achieve a feasible solution and an optimised objective function given the new constraints.

Using Nutrient Reference Values for Australia and New Zealand[29], 20 constraints relating to specific target nutrient densities (nutrient per 1000 kJ) were calculated to achieve the same set of estimated average nutrient requirements (EARs)[28] for a population with the demographic structure of the three communities (Table 3). Nutrient density requirements were therefore expressed per 1000 kJ of the population aggregated energy requirement. Adequate intake (AI) reference values were used for those nutrients where there were no EARs (potassium, dietary fibre and Vitamin E α-tocopherol equivalents)[28]. The upper limit of intake for sodium was used[28]. Additional constraints applied to ensure nutritional adequacy of the community diet were that baseline energy content of the diet was to be maintained, and that the relative macronutrient intake was acceptable (Table 4)[40]. During modelling, the food group nutrient density is a constant value for each of the 34 food groups, however the nutrient density of the overall dietary intake varies with relative contributions of different food groups to the total weight of the diet.

**Table 3:** Nutrient density constraints based on population structure weighted estimated average requirements (EARs), average intakes (AIs) and upper limits (ULs), and relative nutrient densities of Model A and B diets.

Nutrient	Weighted^1^ EAR/1000 kJ^2^ or AI/1000 kJ	Weighted UL/1000 KJ	Nutrient density as a percent of target (%)			
			Baseline intake	Model A	Model B	Model B (UL)
Linoleic (n-6) (g)	1.05 (AI)	-	88.0	106.5	102.5	-
α-Linolenic (n-3) (g)	0.10 (AI)	-	167.8	165.3	167.5	-
LC n-3 (mg)	10.71 (AI)	349.89	119.6	283.4	271.7	8.3
Protein (g)	3.83	-	198.3	263.9	262.0	-
Sodium (mg)	-	224.54	150.7	146.2	148.9	148.9
Potassium (mg)	329.76 (AI)	-	66.0	100.4	100.0	
Iron (mg)	0.72	4.69	202.7	265.8	271.2	41.6
Zinc (mg)	0.76	3.31	126.4	176.4	177.1	40.8
Calcium (mg)	88.42	291.57	71.2	116.9	115.0	34.9
Magnesium (mg)	25.95	31.04	94.1	139.0	138.3	115.6
Phosphorus (mg)	70.56	426.0	175.3	252.1	249.6	41.3
Iodine (*µ*g)	10.33	88.86	109.3	142.1	142.3	16.5
Riboflavin (mg)	0.09		167.5	330.7	328.9	-
Niacin (mg)	1.09	3.03	359.1	455.0	457.5	-
Total folate^3^ (*µ*g)	30.65	85.91	168.2	260.0	212.4	75.8
Vitamin C (mg)	3.35		194.9	293.3	303.0	-
Vitamin A (*µ*g)	52.26	248.99	115.0	237.7	241.3	50.6
Thiamine (mg)	0.09		185.3	291.8	299.0	-
Vitamin E (mg)	0.88 (AI)	25.14	66.1	106.8	102.6	3.6
Dietary fibre (g)	2.687 (AI)		73.7	111.0	113.5	-

^1^ Weighting is based on ABS Census 2006 figures.

^2^ Estimated energy requirements were calculated by age group and sex (1–3 years; 4–8 years; 9–13 years; 14–18 years; 19–30 years; 31–50 years; 51–70 years; >70 years) based on Nutrient Reference Values for Australia and New Zealand, tables 1–3 [28]. For age 19 to >70 years, the midpoint height and weight of each adult age group was used. For <18 years, the midpoint of the estimated energy requirement (BMR) range across each age and sex category was used. Energy expenditure was estimated at 1.6 basal metabolic rate overall. We estimated 8% of women aged 14–50 years were pregnant and 8% were breastfeeding, based on Australian Bureau of Statistics 2006 births data, table 9.2[54] and 2006 census data for women aged 13–54 years.

^3^ EAR and UL for Folate is derived from EAR for Folate as dietary folate equivalents.

**Table 4:** Total (for 3 communities combined for 12 months) edible weight, cost, energy and macronutrient composition for dietary intake of Models A and B compared to baseline.

	Baseline	Model A	Model B	Acceptable range[40]
Cost (million $)	12.6	12.2	11.6	-
		(−3.2%)	(−7.8%)	
Energy Intake (MJ x 1000)	8,878	8,878	8,490	-
		(-)	(−4.4%)	
Protein (% of energy)	12.7	16.7	16.6	15%–25%
Fat (% of energy)	25.7	31.4	30.3	20%–35%
Saturated fat (% of energy)	9.7	12.0	11.7	<10%
Carbohydrate (% of energy)	60.7	52.0	53.2	45%–65%
Sugars (% of energy)	33.4	22.1	22.6	<10%[55]

The guiding principles for the constraints applied to food group intake were: i) no food group would be eliminated; ii) food groups contributing insignificantly to nutrient intake and those foods meeting a specific need and considered to be relatively price inelastic were to be held constant (i.e., artificial sweeteners, cooking additives and infant products); iii) the aggregated weight of “IN” food group categories were to remain constant or increase to no greater than 4 times the baseline measurement (with the exception of the refined cereals food group which was only allowed to decrease and the sweets food group which was not allowed to increase), and “OUT” categories were to remain constant or decrease to no lower than half of baseline measurement (no lower than 10% in the case of refined cereal and beverages); iv) eggs, fruit, legumes, sauces and condiments and wholegrain cereals food groups had only one category and were treated as “IN” with the exception of sauces and condiments which were only permitted to decrease.

The constraints on intake for 34 food groups are shown in Table 2. There were a total of 65 food group constraints (Table 2) and 20 nutrient density parameter constraints (Tables 3 and 4). Optimisation was undertaken using the standard Microsoft Excel Solver software (Frontline Systems Inc, Incline Village, NV). The Microsoft Office Excel Solver tool uses the Generalised Reduced Gradient (GRG2) nonlinear optimisation code, which was developed by Leon Lasdon, University of Texas at Austin, and Allan Waren, Cleveland State University[41].

### Model assessment

The optimised community dietary intake parameters were assessed against: i) population weighted nutrient Upper Limit (UL) recommendations (Table 3)[28]; ii) a maximum intake for red meat of 455grams per person/week[37]; and, iii) the recommended amount in serving sizes for each food group as specified in the AGHE[37] (Table 5). The total number of serves recommended by the AGHE for the study population was estimated by weighting the recommended weekly serves weighted for each age/sex group using the 2006 census data and Omnivore Foundation Diets for boys/girls aged 2–18 yrs; men/women aged 19+ years[37]; and multiplying by total population and 52 weeks/year (Table 5). The number of serves consumed in the baseline community diet, and the optimised diet output were derived by dividing the edible portion weight of appropriate food groups by a reference serve size[37].

**Table 5:** Comparison of recommended food group serves per person per day (Omnivore Foundation diets) to the number of food group serves purchased, Model B.

Food group	Omnivore Foundation diet Recommended weighted number of serves per person per day^1^	Model B number of serves per person per day	Model B as % of recommended weighted intake
Starchy vegetables	0.6	1.2	200
Green and brassica vegetables	0.8	0.4	47
Orange vegetables	0.8	0.3	40
Other vegetables	1.5	1.1	77
Legumes	0.4	0.1	34
Nuts/seeds	0.3	0.1	22
Fruit	1.5	1.0	69
Wholegrain cereals	2.9	2.8	95
Refined cereals	1.4	4.2	295
Poultry, fish and seafood, and eggs	0.7	1.8	236
Red meats	0.7	1.1	152
Dairy foods^2^	2.1	5.8	284
PUS margarine^3^	1.7	1.7	96

^1^ According to the age-groups and gender(37)

^2^ A serving size of 30 g was used representing milk powder which was the dominant food in the dairy food group

^3^ In Model B, the Fats and Oils food group was used to derive number of serves for polyunsaturated margarine (PUS) and a serving size of 10 g was used (representing margarine)

## Results

### Baseline diet

The baseline diet provided an estimated approximate 9200 kJ per person per day (based on the population estimate) at a cost of AUD$13.01 per person per day (Table 4). Details of the baseline diet and population estimate are described elsewhere[1]. In summary, the estimated intakes for 6 out of 20 nutrients (expressed per 1000 kJ) were below recommended levels and sodium was 150.7% of the sodium density upper limit (Table 3). Protein as a percentage of energy was below the recommended range and total sugars as a percentage of energy were more than three times the recommended level (Table 4). Total fat and saturated fat were within the recommended range (Table 4). Refined cereals, sweets and beverages food groups provided 34%, 16% and 12% of energy respectively and contributed 22%, 10% and 25% to diet cost. Table 6 shows the food groups providing nutrients at least cost. The three food groups with the highest sodium density in the baseline diet were cooking additives, sauces and condiments, and sweets (“IN” i.e., artificially sweetened confectionery) and the food groups contributing most to sodium were refined cereals (35.6%), sauces and condiments (18.5%), red meat (“OUT”) (11.2%), and cooking additives (10.5%) (data not presented)[1].

**Table 6:** Food groups providing each nutrient at the least cost^1^.

Nutrient	Food groups providing the nutrient at least cost
Vitamin E	Fats and oils; nuts and seeds
Sodium	Orange vegetables (“OUT”), green and brassica vegetables (“OUT”), fats and oils
Iodine	Eggs, dairy foods
Fibre	Wholegrain cereals, legumes, green and brassica vegetables (“OUT”)
Saturated fat	Fats and oils (“OUT”), fats and oils (“IN”)
Folate	Wholegrain cereals, refined cereals (“IN”)
Vitamin C	Green and brassica vegetables (“IN”), starchy vegetables (“IN”)
Thiamin	Wholegrain cereals, refined cereals (“IN”)
Vitamin A	Orange vegetables (“OUT”), orange vegetables (“IN”), fats and oils (“IN”), fats and oils (“OUT”)
Zinc	Green and brassica vegetables (“OUT”), wholegrain cereals
Iron	Wholegrain cereals, refined cereals (“IN”), green and brassica vegetables (“OUT”)
Magnesium	Wholegrain cereals, nuts and seeds (“IN”), nuts and seeds (“OUT”)
Phosphorus	Wholegrain cereals, dairy foods, eggs
Calcium	Dairy foods
Potassium	Starchy vegetables (“IN”), orange vegetables (“IN”), dairy foods
Sugar	Sweets (“OUT”), beverages (“OUT”)
Protein	Eggs, poultry (“IN”), wholegrain cereals, refined cereals, dairy food
Energy	Fats and oils (“IN”), fats and oils (“OUT”)

^1^ only those food groups for which the total amount is allowed to be modified in the modelling.

### Modelling

A feasible model solution that met all of the desired specifications was not possible. Requirements for sodium and saturated fat could not be achieved. A hierarchy of constraints was therefore developed. First, the food group constraints, energy equality constraint and nutrient density targets (with the exception of sodium) were given highest priority. The constraints for refined cereals and beverages in the “OUT” categories were relaxed to allow these groups to reduce to 0.1 of the baseline value from the specified constraint of 0.5 due to the large proportion of energy these groups contributed. Sodium and macronutrient constraints were treated as secondary priorities as these could not be met simultaneously with the other specifications. Once a feasible solution had been achieved with the revised primary constraints, further modification was undertaken to attempt to achieve targets for sodium and saturated fat. Three models were developed.

Model A was developed to respect all modified specifications. The modelled sodium output was 146.2% of the original constraint and saturated fat contributed 12.0% to energy rather than the recommendation of less than 10% (Tables 3 and 4). Energy was maintained at the observed level and the total diet cost was 3.2% less than the observed expenditure. Model B was developed to extend Model A to reduce sodium and saturated fat (Tables 3 and 4). The energy constraint was relaxed to ±5% of the baseline value, to allow the model more flexibility. Targets for sodium (148.9%) and saturated fat (11.7%) were still not achieved. Energy intake was lowered by 4.4% and cost was 7.8% less than baseline expenditure. Both Models A and B exceeded the population weighted upper limit nutrient density target for magnesium (Table 3) – however as far as we know the upper limit for magnesium applies only to magnesium in supplements – and no toxic effect of magnesium naturally occurring in food has ever been shown[28].

It was possible to achieve the dietary saturated fat target by a modification to the “IN” and “OUT” composition of the food groups used in Model B (Model B variation). A large percentage of dietary saturated fat (43%) was provided from the dairy food group “OUT” category in Model B, which was the category contributing most to total dairy food intake. When the dairy food group was modified so that dairy “IN” foods (i.e., reduced fat dairy foods) contributed 70% to the food group weight and dairy “OUT” foods only 30%, the resulting model provided a combination of food groups that achieved the saturated fat intake constraint of below 10% of energy. This change to the type of dairy food intake was a large change to the baseline diet and reduced energy intake beyond the energy constraint.

To explore if the sodium density target could be met at all under the given food groupings and food group constraints, all nutrient density constraints were removed one by one, however the sodium density constraint could still not be achieved. Further, all food groups with a sodium density below and above the sodium constraint were identified and taken to their maximum and lower limit respectively. This Model (Model C) failed to meet the sodium density target or many of the other nutrient density targets, and dietary energy intake was excessively raised.

The most feasible models (A and B) indicate that the dietary changes required to achieve nutrient adequacy at minimal cost are substantial (Table 7). As shown in Table 7, Models A and B require an approximate four-fold increase in eggs, fruit and wholegrain cereals and the “IN” sub-categories of dairy, fish and seafood and all vegetable groups, and; a large reduction in beverages (to 50% and 10% of the baseline beverage intake for “IN” and “OUT” beverages respectively) and refined cereals (to 69% and 10% of baseline intake for “IN” and “OUT” groups respectively).

**Table 7:** Optimised food group intake compared to baseline, Models A and B.

Food group	Baseline	Model A	Model B
	(g x 1000 kg)	% of baseline	% of baseline
Artificial sweeteners	0.03	100	100
Beverages (“IN”)	830	50	50
Beverages (“OUT”)	580	10	10
Cooking additives	0.58	100	100
Dairy Foods (“IN”)	8.16	400	371
Dairy foods (“OUT”)	78.4	200	184
Eggs	25.2	400	380
Fats and Oils	15.7	100	100
Fats and Oils (“OUT”)	1.14	50	50
Fish and Seafood (“IN”)	12.4	400	354
Fish and seafood (“OUT”)	0.16	98	50
Fruit	45.1	400	400
Green and brassica Vegetables (“IN”)	7.98	400	400
Green and brassica Vegetables (“OUT”)	0.04	100	100
Infant products	0.88	100	100
Legumes	9.04	100	100
Nuts and seeds (“IN”)	0.70	400	100
Nuts and seeds (“OUT”)	3.00	99	50
Orange Vegetables (“IN”)	6.87	400	400
Orange vegetables (“OUT”)	0.10	100	100
Other vegetables (“IN”)	21.3	400	400
Other vegetables (“OUT”)	2.15	100	50
Poultry (“IN”)	15.5	100	100
Poultry (“OUT”)	19.6	100	76
Red Meat (“IN”)	38.3	100	100
Red meat (“OUT”)	75.9	50	50
Refined Cereal (“IN”)	228	70	69
Refined cereals (“OUT”)	39.5	10	10
Sauces	8.34	100	100
Starchy vegetables (“IN”)	21.6	400	400
Starchy vegetables (“OUT”)	14.7	100	62
Sweets (“IN”)	2.49	100	100
Sweets (“OUT”)	95.1	50	50
Wholegrain cereals	27.0	400	400

In comparing the optimised diets (Model B is used to demonstrate this) to food intake recommended by the AGHE, there are large disparities (Table 5): refined cereals and dairy foods are almost 3 times higher, starchy vegetables and poultry/fish/eggs are twice that recommended, and red meat is 50% higher than that recommended. All other vegetables, legumes (34%), fruit (69%), nuts and seeds (22%) are provided in much lower quantities than that recommended (Table 5).

## Discussion

This study was able to identify a dietary pattern that addressed national nutrient recommendations for approximately AUD$12.59/person/day. It provides evidence that a route for remote Aboriginal communities to meet their nutrient requirements, at lowest cost, is a combination of: increasing intake of most categories of vegetables, fruit, fish, eggs, dairy food and wholegrain cereals, and markedly reducing intake of beverages and refined cereals.

From the perspective of prevention of diet-related diseases that are excessive among Aboriginal Australians and reducing the disparity in life expectancy between Indigenous and non-Indigenous Australians, there are substantial benefits in such a dietary pattern. This finding supports other studies that have also found that a healthier diet is not necessarily a more expensive diet[14];[15]. However this dietary pattern cannot be achieved at much less than current food spending which is already higher than that of the rest of Australia, due to higher food costs in remote Australia[1].

Our modelling approach showed that it was not feasible to achieve all nutrient targets under the current food supply with a dietary pattern resembling current food intake. Despite the large dietary shifts required to meet most nutrient targets, sodium intake would remain 1.5 times the recommended upper limit and saturated fat above the recommended level. Switching the type of sauces and condiments and dairy foods consumed (for example using low salt and reduced fat products) would have an impact on salt and saturated fat intakes, but would incur an added cost. This was demonstrated by the Model B variation where the saturated fat target was achieved by modifying the dairy food group to mostly reduced-fat dairy food, but this reduced energy intake to below specifications, requiring additional expenditure to replace it. Other investigators have observed similar outcomes for sodium, concluding that the extent to which salt is added to food in the modern food supply makes limiting sodium intake to the recommended level while simultaneously achieving requirements for all other nutrients impossible[42]; [43]. For example, despite its high sodium density, the sauces and condiments group did not decrease from the observed weight in the modelled diets presumably due to its high iodine density. Guenther et al (2013) recently demonstrated that nutritionally adequate diets, based on the dietary guidelines for Americans, could be constructed by allowing only low-sodium foods, but concluded that such diets would be very challenging[44]. Since the harmful effects of consuming too much sodium are well established[45], this study also highlights that it is imperative to take a population level approach to reduce the amount of sodium used in food manufacturing and processing, while also encouraging consumers to seek foods within categories that are lower in salt (or sodium) and to use less salt in food preparation and less table salt.

Although most nutrient recommendations were achieved, the modelled diet does not match the AGHE recommendations. The modelling processes used in this study and for AGHE are designed to arrive at recommendations that achieve nutrient targets and are consistent with the eating patterns of the target population. A key difference to our modelling approach was that the AGHE optimisation modelling was based on achieving RDIs (which are higher than EARs), and did not include as modelling criteria those nutrients where requirements are described by AIs only. Further, the modelling of this study was based on a total self-selected diet whereas the AGHE modelling for the foundation diets did not include discretionary foods (food and drinks generally less nutrient dense and higher in fats, added sugars, salt and alcohol) and was designed to provide nutrient requirements at least energy rather than at least cost[37]. A further explanation for the modelled diet of this study not matching AGHE recommendations is that the baseline dietary pattern of remote Australian Indigenous communities is very different to the dietary pattern for the general Australian population. For example, the relatively low base of fruit and vegetable consumption means that even when intake is increased by four times, the total intake still does not reach the AGHE recommendations.

Limitations in this modelling approach need consideration. It is assumed in this study, that food is distributed within the community according to nutrient need; that food purchase data are complete; that there is minimal wastage after purchasing food; and that the population structure used was correct for the period of measurement. The study accounts for population heterogeneity by determining average nutrient requirement levels based on the age and gender distribution of the population, however it does not account for differing activity levels or body sizes[46]. The study does not take into account food sourced outside of the community store/s and food services. The modelling activity however deals with the cost and nutritional quality of a fixed large proportion of the total community food supply which means that the results are broadly applicable to total food intake. Although the level of energy provided through the purchased food supply suggests minimal contribution of traditional foods to energy intake, more research is warranted on the nutritional contribution of traditional foods considering their high relative nutrient density compared to purchased foods[1].

Minimal cost was the primary objective function of the modelling, however, the constraints applied to the food groups to minimise deviation from the baseline diet, had a greater impact on the modelling output than differences in cost between food groups. For example, for each of the vegetable groups, the modelling increased the “IN” category to the highest level allowed (up to 4 times); whereas, refined cereals were forced to reduce. A further example concerns the dairy food group where because of the large difference in baseline volume between the “IN” and “OUT” categories, the dairy “OUT” was able to increase by nearly ten times that of the “IN” category, as both categories could increase up to four times. These constraints however helped to minimise departure from the current diet, providing food intake patterns that may be pragmatic despite the large changes. It is important to note that the components of the composite food groups in the modelled diet reflect that of the baseline diet.

Relationships between food groups are also difficult to deal with in modelling. For example, there was a substantial decline in refined cereals (e.g., bread and flour), and less variation in fats and oils (e.g., margarine) and cooking additives (e.g., baking powder), which are generally used in association with bread and flour.

This is the first study we know of that has attempted to estimate the cost of dietary improvement for a population based on entire purchased food data records. Strengths of this study are that it has i) used measurement of the actual community level diet; ii) modelled a recommended diet based on estimated requirements for a large number of specific nutrients and other dietary intake parameters; and, iii) used total community-level food purchase data collected for a twelve month period. The use of food purchase data as a direct and objective measure of community level dietary intake overcomes some methodological problems inherent in dietary surveys[47]. This study reports on the cost of dietary improvement at least cost for three remote Aboriginal populations. The nutrient profile for one community was worse than the other two and dominated the total community diet due to a large population size[1]. However these three communities represent the variation that exists in relation to size, remoteness and number of food businesses and services across remote Aboriginal communities. Similar nutrition profiles and socio-economic conditions and disparities in the cost of food exist across Aboriginal communities in remote Australia[4]; [6]. Applying this technique of optimisation modelling over a larger number of communities however is warranted.

### Possible Policy Implications

These results could help guide local and government policies in considering strategies for dietary improvement in remote Australia and possible cost implications. For example, the only cost considered in the modelling was the purchase cost of food in the community. As the modelling shifts significant amounts of processed “long-shelf life” convenience foods to minimally processed foods, which have higher costs associated with freight, storage and food preparation and wastage, additional retailer and therefore consumer costs to achieve dietary improvement are likely. Freight subsidies and provided capital for storage and preparation facilities might alleviate the need for these imposed costs. As there are no immediate cost savings to the consumer in switching to a healthy diet, the costs of shifting individual eating behaviour from the current diet need to be considered. The ability for home food preparation in remote Aboriginal communities is severely compromised by many factors including over-crowded housing, inadequate food preparation and storage facilities, the high cost of food, and the high costs of cooking equipment, power and appliances such as refrigerators[48]. These in turn result in the lack of opportunities for young people to learn cooking skills in the home through observation of their parents and elders. A focus on enhancing basic food preparation and cooking skills of young people and initiatives to support household purchase of whitegoods such as refrigerators could help address this. Shifting likely entrenched perceptions that a healthier diet would cost more than the current diet would further require innovative strategies.

Increasing attention is being given to the use of economic incentives (i.e., taxation, subsidies or direct pricing) to modify individual dietary behaviour. While taxes on sugar sweetened beverages and fat taxes continue to be considered by public health policy makers to modify eating behaviour and curb obesity, there is little evidence to date of their effect[49] and concerns about their potentially regressive nature[50]. This study suggests that a fat tax to lower intake of saturated fat would not be relevant for remote Aboriginal communities as the baseline intake is relatively low. There is evidence from some general population studies that subsidising healthier foods can modify dietary behaviour[51]. There is little such evidence relating specifically to socio-economically disadvantaged populations[51]. The US Department of Agriculture currently has a pilot project in place to examine the effectiveness of a 30% discount on fruit and vegetables, applied through a reward-type program, in changing dietary behaviour among low income residents enrolled in the Supplemental Nutrition Assistance Program[52]. Customer loyalty programs appear to be promising and acceptable in delivering incentive schemes to promote healthy eating[53]. The acceptability and feasibility of such programs needs to be evaluated in the remote Aboriginal context.

In conclusion, complete food purchase data at a population level can be used to estimate the dietary change required to achieve nutrient recommendations at minimum cost. Our results show that large shifts in the diet in the study communities are needed to achieve national nutrient recommendations (excluding sodium and saturated fat targets) without the possibility of substantial food cost savings. In the current food environment, dietary improvement requires a switch from highly processed foods and sugar sweetened beverages to minimally processed foods. As there are no immediate savings in eating healthily, financial incentives and political commitment will be required to support dietary improvement.
